# KIF20A is associated with clinical prognosis and synergistic effect of gemcitabine combined with ferroptosis inducer in lung adenocarcinoma

**DOI:** 10.3389/fphar.2022.1007429

**Published:** 2022-09-26

**Authors:** Hua He, Lu Liang, Jingjing Huang, Shiyao Jiang, Yueying Liu, Xiaoyan Sun, Yi Li, Li Cong, Yiqun Jiang

**Affiliations:** ^1^ The Key Laboratory of Model Animal and Stem Cell Biology in Hunan Province, Hunan Normal University, Changsha, Hunan, China; ^2^ School of Medicine, Hunan Normal University, Changsha, Hunan, China

**Keywords:** lung adenocarcinoma, gemcitabine, ferroptosis, synergistic effect, risk model, KIF20A

## Abstract

Gemcitabine (GEM), an antimetabolite that terminates DNA synthesis, is commonly used in the treatment of cancers including lung adenocarcinoma (LUAD). However, downregulation of sensitivity limits the therapeutic effect. Ferroptosis as the new form of regulated cell death has been shown to have great potential for cancer treatment with chemoresistance. Here, three genes with both ferroptosis and GEM-response-associated features were screened from RNA sequencing and public data for constructing an independent risk model. LUAD patients with different risk scores had differences in mutational landscape, gene enrichment pathways, and drug sensitivity. By Cell Counting Kit-8 assay, flow cytometry, and colony forming assay, we demonstrate that GEM and ferroptosis inducer (FIN) imidazole Ketone Erastin had a synergistic combined anti-proliferative effect on LUAD cells and knockdown of *KIF20A* (the core gene of our model) further enhanced cell death *in vitro* by inducing ferroptosis. In conclusion, we identified a link between ferroptosis and GEM response in LUAD cells and developed a robust signature that can effectively classify LUAD patients into subgroups with different overall survival. For LUAD, the combined treatment modality of GEM and FIN is potentially effective and *KIF20A* may be a new therapeutic target.

## Introduction

Lung cancer remains the leading killer of cancer deaths in both men and women (Siegel et al., 2022), and it is mainly divided into small cell lung cancer and non-small cell lung cancer (NSCLC), while lung adenocarcinoma (LUAD) accounts for 78% of NSCLC (Thai et al., 2021). Poor survival in LUAD is partly attributed to the fact that some LUAD patients have been diagnosed at distant metastatic stages or late stage. For advanced treatment, despite the greater advances in the use of targeted therapies for specific genetic mutations and immune checkpoint inhibitors, more patients benefit from a combination therapy approach with platinum, gemcitabine, paclitaxel, docetaxel and other drugs (Miller and Hanna, 2021; Thai et al., 2021).

Gemcitabine (GEM), a structurally similar compound to cytarabine, is converted intracellularly to the nucleotides gemcitabine diphosphate and triphosphate, the latter causing DNA chain termination and thus interfering with DNA synthesis (Mini et al., 2006). Early clinical trials have demonstrated that GEM is considered effective in the treatment of advanced NSCLC, both alone and in combination with Cisplatin (Mosconi et al., 1997; Sandler and Ettinger, 1999), and not only that, but GEM is also an effective radiosensitizer (Mornex and Girard, 2006). It is important to mention that intrinsic and acquired drug resistance has made GEM less effective for cancer treatment. Some mechanisms related to GEM resistance have been disclosed in recent years. For example, mTORC2 helps NSCLC cells evade GEM-induced cell death by regulating Ribonucleotide reductase activity and DNA replication (Tian et al., 2021). Coordinated metabolic reprogramming of HIF-1α/ABCB6 promotes reactive oxygen species (ROS) clearance directly contributing to the inhibition of apoptosis in tumor cells and the promotion of GEM resistance in NSCLC cancer (Xiang et al., 2022). Hence, exploring effective cell death may be beneficial to address the resistance of LUAD to GEM.

Ferroptosis, characterized by the iron-dependent accumulation of lipid ROS, is a non-apoptotic regulated cell death (RCD) that has received the most attention in recent years (Dixon et al., 2012). Ferroptosis has created new opportunities for the treatment of cancers that are not sensitive to chemotherapeutic agents (Friedmann Angeli et al., 2019; Lei et al., 2022). Notably, the use of experimental compounds such as erastin and RSL3, which can act as ferroptosis inducers (FINs) significantly enhanced the effects of some anticancer drugs. A study reported that suppression of cystine/glutamate antibody solute carrier family 7 member 11 (*SLC7A11*) by erastin and sulfasalazine to promote ferroptosis increased the sensitivity of drug-resistant head and neck cancer cells to Cisplatin (Roh et al., 2016). The combined treatment of Cisplatin and erastin also has a significant synergistic effect on proliferation inhibition in NSCLC cells (Guo et al., 2018). In addition, several FDA-approved drugs can also induce ferroptosis in cancer cells (Hassannia et al., 2019; Chen et al., 2021a). Ferroptosis inhibitors can partially block the cytotoxicity of sorafenib in Hepatocellular carcinoma (HCC) cells, suggesting that the anticancer effect of Sorafenib may be partially mediated by ferroptosis (Louandre et al., 2013). In the treatment of Pancreatic adenocarcinoma (PAAD), it has been reported that induction of ferroptosis may down-regulate GEM resistance (Yang J. et al., 2021). As a result, ferroptosis may play an active role in regulating GEM response in LUAD cells, and the identification of genes regulating GEM response and ferroptosis is important for investigating new therapeutic targets.

In this study, we identified ferroptosis-related genes (FRGs) which also regulated GEM response and constructed a prognostic model for clinical patient samples that showed high diagnostic accuracy and correlated with mutational landscape and drug sensitivity. Core genes *KIF20A* of the model may have a role in mediating LUAD sensitivity to GEM and the combination of GEM and IKE synergistically induced LUAD cell death *in vitro*. Our study may provide new molecular and therapeutic strategies for the treatment and prognosis of LUAD patients.

## Materials and methods

### Cell culture

Human embryonic kidney 293T cells, human bronchial epithelial-like cells 16HBE and LUAD cells (H358, PC9, HCC827, H1299, A549) were obtained from ATCC. 16HBE and 293T cells were maintained in DMEM (Gibco, Carlsbad, CA, United States); A549 cells were maintained in DMEM/F12 1:1 (Gibco, Carlsbad, CA, United States); other cells were maintained in RPMI 1640 (Gibco, Carlsbad, CA, United States). All media were supplemented with 10% fetal bovine serum (FBS, Gibco, Carlsbad, CA, United States), 100 mg/ml penicillin, and 100 mg/ml streptomycin solution. All cell lines are maintained at 37°C with 5% CO_2_ and tested negative for *mycoplasma* contamination.

### RNA sequencing (RNA-seq)

A549 cells were treated with 20 nM GEM or PBS for 48 h. The cells were washed one time with pre-cooled PBS and lysed with 1 ml Total RNA Extraction Reagent (TRIzol, TR401-01, Vazyme, Nanjing, China). Total RNA was collected and sent to Shanghai Majorbio Bio-pharm Technology Co., Ltd. (Shanghai, China) for RNA expression profiling. The differentially expressed genes (DEGs) between the GEM-treated groups and control groups were screened using the “edgeR” R package (|FC|>1.2, *p* < 0.05).

### Acquisition of public data

RNA-seq of 51 LUAD cell lines and half maximal inhibitory concentration (IC50) values for various drugs were downloaded from the Genomics of Drug Sensitivity in Cancer (GDSC, https://www.cancerrxgene.org/). Gene expression data and corresponding clinical information of LUAD patients and PAAD patients were obtained from The Cancer Genome Atlas (TCGA, https://cancergenome.nih.gov/) and Gene Expression Omnibus (GEO, http://www.ncbi.nlm.nih.gov/geo), and patients with one or more unavailable clinical features were excluded. A list of recognized FRGs was collected from FerrDb (http://www.zhounan.org/ferrdb/current/). DEGs were analyzed using the R package “limma.” RNA-seq data of normal lung tissue was downloaded from Genotype-Tissue Expression (GTEx, https://www.gtexportal.org). Immunohistochemical (IHC) staining data of KIF20A protein expression and distribution in LUAD tissues were obtained from the Human Protein Atlas (HPA) (Uhlen et al., 2015; Uhlen et al., 2017) (https://www.proteinatlas.org/).

### Identification of genes associated with GEM response and ferroptosis

DEGs from A549 cells RNA-seq and DEGs from GDSC were cross-tabulated using the “Venn Diagram” R package to obtain genes associated with GEM response. Multifactorial Cox regression analysis of 264 ferroptosis driver genes and 240 suppressor genes (FRGs) in the TCGA-LUAD (*n* = 345) data and GSE97489 dataset (*n* = 393) was performed separately. Prognostic genes which were screened by hazard ratio (HR) > 1 were again intersected with GEM sensitivity-related genes, and the final candidate genes were identified. The “survival” “ggplot2” “vioplot” R package is used to present the connection between candidate gene expression and patient overall survival (OS), tumor stage and gene comparative expression in normal and tumor samples, respectively.

### Construction and validation of model

The risk score for each patient was calculated using the formula: risk score = sum (each candidate gene expression × Multivariate Cox regression coefficient). From this, we ranked patients according to the risk score, with median groupings generating a low-risk group and a high-risk group. The R package “ggrisk” is utilized for risk plots. The heat map was completed using the “pheatmap” R package and shows the expression levels of candidate genes in the high-risk score group and the low-risk score group. The R packages “stats” “Rtsne”, “umapr”, and “ggbiplot” were used to genes downscale and visualize results of principal component analysis (PCA), t-distributed Stochastic Neighbor Embedding (t-SNE), and uniform manifold approximation and projection (UMAP), showing the distribution of different groups. To explore the difference in survival between high-risk and low-risk patients, “survival” R package was used for Kaplan-Meier (K-M) survival analysis. The “rms” R package is used to plot the nomogram which pooled model score, age, and tumor stage to calculate patient survival probability at 1, 3, and 5 years. Calibration curves are drawn to evaluate the accuracy of model. The “survival ROC” package (Heagerty and Zheng, 2005) plots the receiver operating characteristic (ROC) curves while calculating the area under the curve (AUC) at 1-, 3- and 5-year, respectively, confirming the reliability of the risk model.

### Somatic mutation analyses and gene set enrichment analysis

The R package “maftools” is used to plot a waterfall of the mutation landscape, showing the genes with the highest mutation frequency (Top10). The DEGs of the high-risk and low-risk groups were screened using the “limma” R package (|FC|>1.5, *p* < 0.05). The Gene Ontology (GO) and Kyoto Encyclopedia of Genes and Genomes (KEGG) pathway for DEGs were used for enrichment analysis, which was performed using the “clusterProfiler” R package (Yu et al., 2012) and the results were plotted using the “ggplot2” R package for bubble and bar plots. Gene set variation analysis (GSVA) was performed based on the “GSVA” R package (Hanzelmann et al., 2013) and gene sets to measure the signaling pathway variation scores of each sample in different groups, with heatmap visualized using the “pheatmap” R package.

### Drug sensitivity analysis

IC50 values for each drug in LUAD cell lines were derived from GDSC and the risk score of each LUAD cell line was calculated using the same formula as above: risk score = sum (each candidate gene expression × Multivariate Cox regression coefficient). The association of these IC50 values with risk scores, and candidate gene expression levels was interpreted using the “pRRophetic” “ggplot2” “vioplot” R packages.

### Plasmids and lentiviral infection

To generate LUAD cells with knockdown *KIF20A*, we purchased *KIF20A*-shRNA as well as a scramble control vector from Genechem (https://www.genechem.com.cn; Shanghai, China). All constructs were confirmed by DNA sequencing. The target sequences used are as follows:

shCtrl: 5′- GCA​AGC​TGA​CCC​TGA​AGT​TCA-3′;

sh*KIF20A*#1: 5′-CCT​GAA​GAA​ATA​GGT​CTC​TTT-3′;

sh*KIF20A*#2: 5′-CCG​ATG​ACG​ATG​TCG​TAG​TTT-3′;

sh*KIF20A*#3: 5′-CCG​TTC​CTG​CAT​GAT​TGT​CAA-3′.

shCtrl/sh*KIF20A* plasmid and lentiviral packaging plasmid were transfected into 293T cells, and the virus supernatant was collected after 48 h, filtered through a 0.45 μm filter, and then infected with LUAD cells together with 1 μg/ml polybrene (C0351, Beyotime, Shanghai, China) and incubated for 48 h. The virus solution was replaced with fresh medium containing puromycin (ST551, Beyotime, Shanghai, China) for screening, and the final LUAD cell line with stable knockdown of *KIF20A* was obtained.

### Western blot analysis

Detailed information about the Western blot analysis was previously described (Liang et al., 2022a). The antibodies used are as follows: β-actin antibody (AF7018, Affinity), KIF20A antibody (AF7664, Affinity), Goat Anti-Rabbit IgG (H + L) HRP (S0001, Affinity) and Goat Anti-Mouse IgG (H + L) HRP (S0002, Affinity).

### Reverse transcription quantitative real-time PCR (RT-qPCR)

TRIzol was used to isolate total RNA, and HiScript® II Q RT SuperMix for qPCR (+gDNA wiper) kit (R223, Vazyme, Nanjing, China) was used to generate cDNA. The Bio-Rad CFX Connect Real-Time PCR System with MonAmp™ ChemoHS qPCR Mix (MQ00401S, Monad, Shanghai, China) was used to perform real-time PCR for detecting triplicate samples (Liang et al., 2022b). Normalization of relative gene expression was achieved by β-actin.

The *KIF20A* primers were designed as follows:

Forward Sequence: 5′- CAA​GAG​GCA​GAC​TTT​GCG​GCT​A -3′;
Reverse Sequence: 5′- GCT​CTG​GTT​CTT​ACG​ACC​CAC​T -3′.

The β-actin primers were designed as follows:

Forward Sequence: 5′-CAC​CAT​TGG​CAA​TGA​GCG​GTT​C-3′;
Reverse Sequence:5′-AGGTCTTTGCGGATGTCCACGT -3′.

### Cell Counting Kit-8 assay

The LUAD cell line A549/PC9 was seeded at 2000 cells per well in 96-well plates and incubated overnight to allow cell attachment. Medium containing GEM (S1149, Selleck) or/and imidazole Ketone Erastin (IKE, S8877, Selleck) were added to the indicated wells and after 48 h incubation 100 µl of mixed medium containing 10% Cell Counting Kit-8 (CCK-8, A311-01, Vazyme, Nanjing, China) was added to each well and incubated for 2 h at 37°C, protected from light, followed by measurement of the absorbance of each sample at 450 nm using an enzyme marker (Biotek, United States) (Huang et al., 2022). The data were analyzed statistically using GraphPad Prism 8.02. The combination index (CI) of GEM and IKE was calculated using CompuSyn (Cambridge, United kingdom). CI = 1 or >1 indicates additive and antagonistic effects, respectively, while CI < 1 indicates synergistic effects.

### Colony-forming assay

Approximately 500 LUAD cells were seeded into 6-well plates and cultured for 24 h, then cells were treated with either DMSO, 20nM/1000 nM GEM, 2 μM IKE or combination for 12 days. Cells were fixed in methanol for 15 min and stained with 0.1% crystal violet for 20 min (Liang et al., 2022a). Microscopy and ImageJ software were used to visualize and count the number of clones.

### Lipid ROS measurement

For the lipid ROS assay, details were as described previously (Jiang et al., 2017). Briefly, cells were treated with IKE (5 μM) for 24 h and then resuspended in medium, followed by addition of 10 μM C11-BODIPY (Thermo Fisher, Cat# D3861) for 30 min under light-protected conditions. Cells were washed twice with PBS and then analyzed for lipid ROS production using a flow cytometer (FACSCCantoII, BD, United States).

### Statistical analysis

All statistical analyses and plots were performed using R software (version 4.2.0) or GraphPad Prism (version 8.02). Comparisons between two groups were analyzed using Student’s *t*-test or Wilcoxon signed-rank test. Multivariate Cox regression analysis was performed to identify independent prognosis of FRGs. Spearman correlation analysis was used for correlation analysis between risk scores and drug IC50. Two-tailed *p* < 0.05 was considered statistically significant.

## Results

### Three genes were screened for prognostic FRG concerning GEM response

**FIGURE 1:**
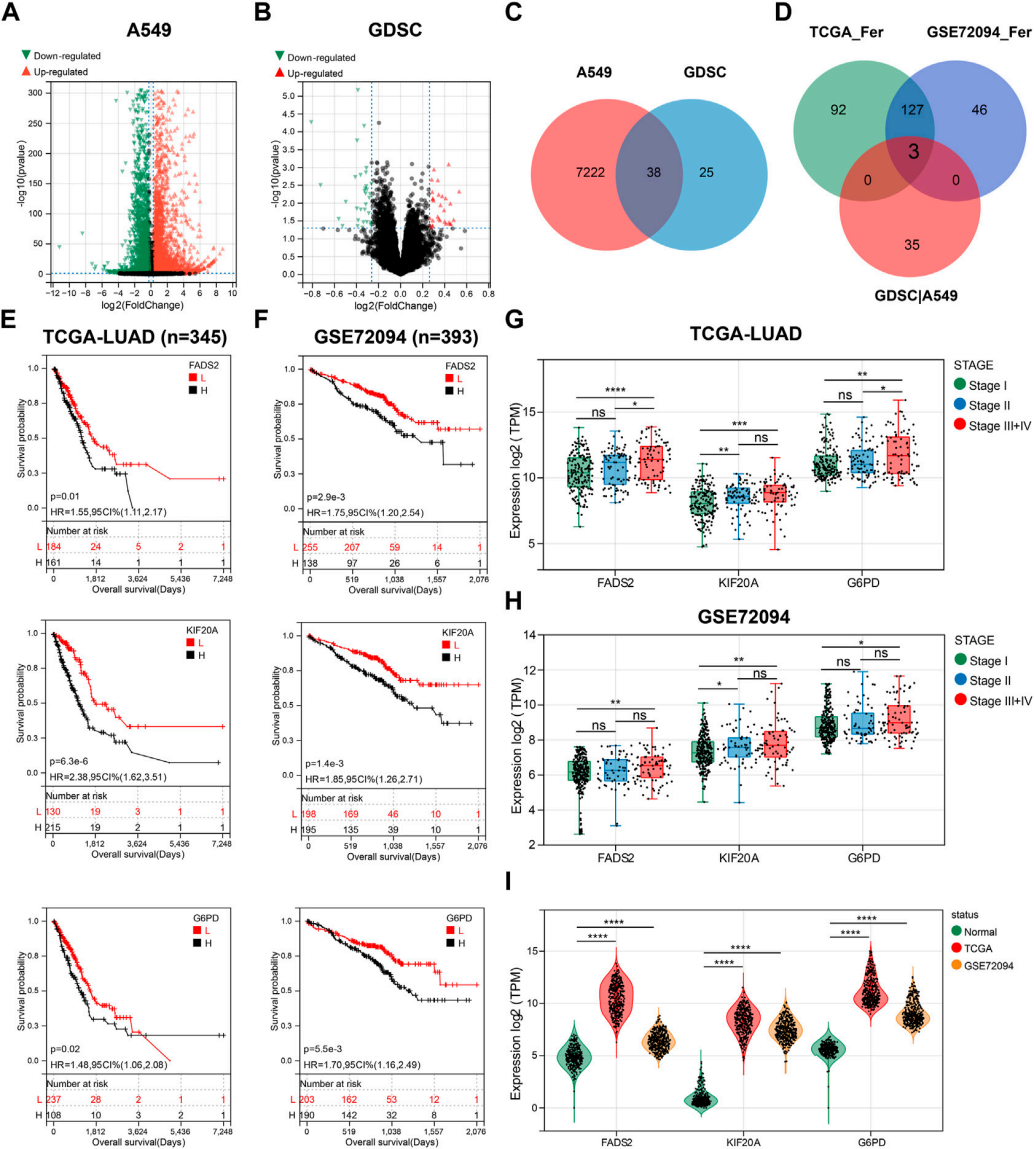
Three GEM response-related prognostic FGRs were screened. **(A)** A volcano plot of data obtained by RNA-seq comparing A549 cells exposed to GEM and PBS for 48 h. **(B)** The volcano map showing the DEG between non-sensitive LUAD cells and GEM-sensitive LUAD cells. **(C)** Venn diagram depicting 38 screened GEM response-related genes. **(D)** Venn diagram to identify the common FRGs of GEM response-related genes, TCGA-LUAD prognostic FRGs and GES72094 prognostic FRGs. **(E,F)** K-M curves for OS of LUAD patients with high and low expression groups of the three candidate genes. **(G,H)** Box plots showed the expression of three candidate genes in different tumor staging samples. **(I)** Violin plots for comparing the expression levels of three candidate genes in normal lung tissue and LUAD samples. FRGs: ferroptosis-related genes; GEM: Gemcitabine; TCGA_Fer: prognostic FRGs in TCGA-LUAD; GSE72094_Fer: prognostic FRGs in GSE72094; GDSC|A549: the intersection genes of DEGs in GEM-treated A549 and GDSC databases; RNA-seq: RNA sequencing; LUAD: lung adenocarcinoma; K-M analysis: Kaplan-Meier analysis; OS: overall survival; HR: hazard ratio. *p < 0.05; **p < 0.01; ***p < 0.001; ****p < 0.0001; ns *p* > 0.05.

To explore the link between GEM and ferroptosis, we used RNA-seq and bioinformatics for analysis. 7260 DEGs of A549 treated with GEM (20 nM) for 48 h versus control cells were presented using volcano plots (Figure 1A, |FC|>1.2, *p* < 0.05). 51 LUAD cell lines are ranked according to their GEM IC50 values from highest to lowest, with the top ten cells with IC50 values being considered relatively insensitive and the bottom ten cell lines being relatively sensitive. A volcano plot was drawn based on RNA-seq of these cell lines, showing the 63 DEGs of cells in the relatively insensitive group versus those in the sensitive group **(** Figure 1B, |FC| >1.2, *p* < 0.05). The DEGs of the two datasets were intersected and 38 genes were considered to be related to GEM response (Figure 1C). 222 and 176 prognostic FRGs (HR > 1) were subsequently identified in the TCGA-LUAD and GSE97489 cohorts, respectively, by multivariate Cox regression analysis. The table demonstrates the top 15 drivers or suppressors of ferroptosis by HR (Supplementary Table S1). Finally, we found three genes (*FADS2*, *KIF20A* and *G6PD*) present in the prognostic FRGs of TCGA-LUAD cohort and GSE97489 cohort, as well as associated with GEM response (Figure 1D). Based on the optimal truncation grouping, patients in TCGA-LUAD and GSE97489 were, respectively classified into *FADS2* high and low expression groups. We noticed that the OS of the high expression group of *FADS2* was significantly shorter than that of the low expression group, suggesting that *FADS2* high expression was associated with poor prognosis of patients, and the same results were observed for *KIF20A* and *G6PD* (Figures 1E,F). GEM is mostly used for the advanced treatment of LUAD, and we found expression levels of the three prognostic genes were higher in LUAD stage III and IV than in stage I (Figures 1G,H **)**. The results of tumor-node-metastasis (TNM) staging analysis also indicated that the expression of three prognostic genes was higher in N2 than in N0 stage (Supplementary Figure S1). In addition, *FADS2*, *KIF20A* and *G6PD* were all highly expressed in LUAD compared to normal lung tissue (*n* = 288, Figure 1I). Therefore, we screened for three GEM-response-associated prognostic FRGs, which were not only highly expressed in LUAD but also associated with shorter OS, and higher tumor stage in LUAD patients.

### GEM response and ferroptosis related model was constructed and validated

**FIGURE 2:**
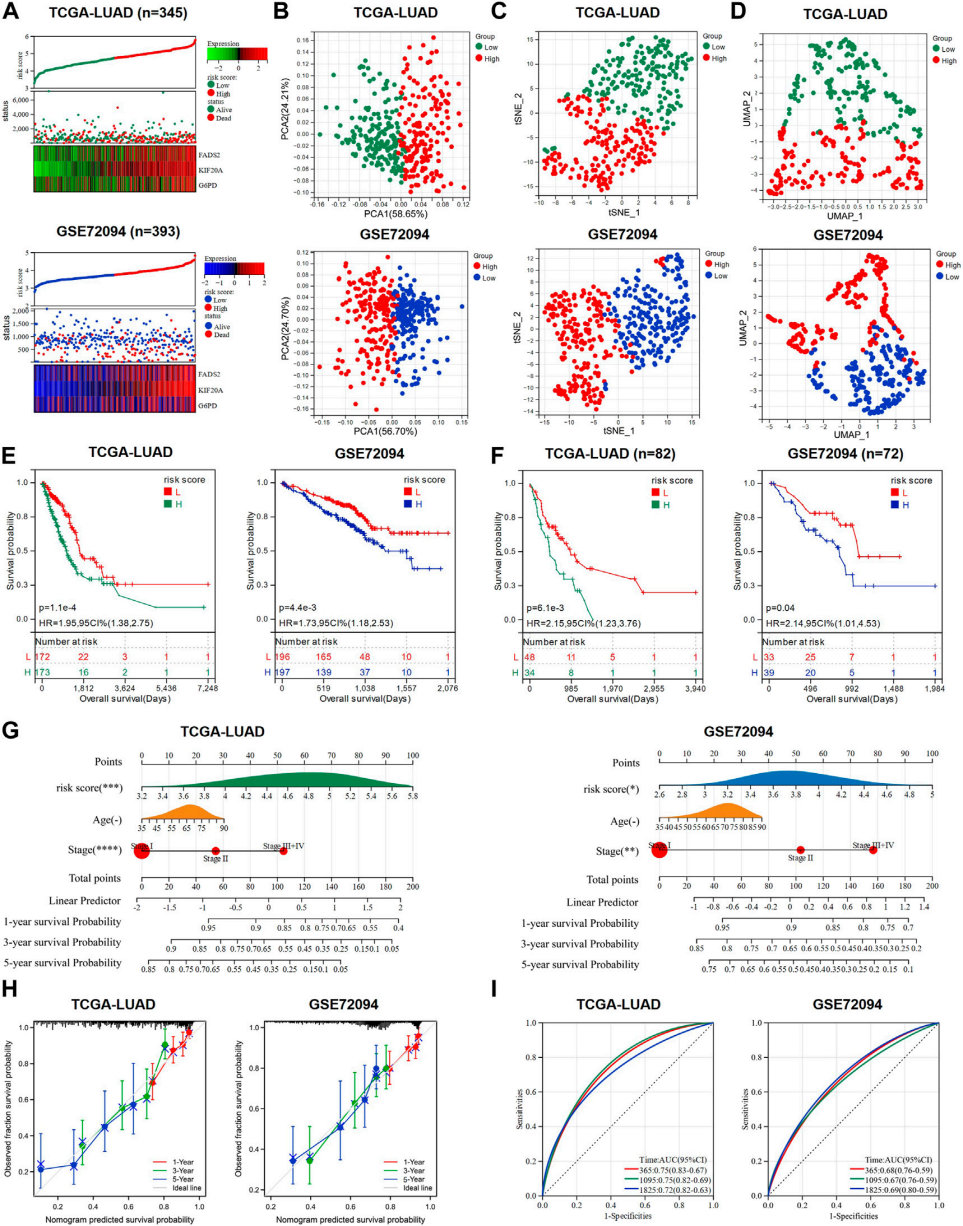
A prognostic model to predict the survival of LUAD patients was constructed and validated. **(A)** Expression heat map of the three candidate genes, risk score curve and survival status scatter plot for each LUAD patient in training cohort and validation cohort, respectively. **(B–D)** PCA analysis, t-SNE analysis and UMAP analysis were used to verify the grouping performance of the prognostic model. **(E)** K-M curves for OS of LUAD patients in the high-risk and low-risk groups. **(F)** K-M curves for OS of advanced LUAD patients (stage III + IV) in the high-risk and low-risk groups. **(G)** Nomograms were constructed using three independent prognostic factors (risk score, age, and tumor stage) to predict OS at 1-, 3-and 5-year for LUAD patients. **(H)** The calibration plots assess the accuracy of the nomogram. **(I)** AUC of ROC curves for validating the accuracy of risk model 1-, 3- and 5-year survival predictions. LUAD: lung adenocarcinoma; TCGA: The Cancer Genome Atlas; PCA: principal component analysis; t-SNE: t-distributed Stochastic Neighbor Embedding; UMAP: uniform manifold approximation and projection; K-M analysis: Kaplan-Meier analysis; OS: overall survival; ROC: receiver operating characteristic; AUC: area under the curve; HR: hazard ratio. *p < 0.05; **p < 0.01; ***p < 0.001; ****p < 0.0001; ns *p* > 0.05.

We adopted TCGA-LUAD as the training cohort and GSE97489 as the validation cohort for the analysis of the prognostic model and named this model as GEM response and ferroptosis related model (GRFRM). Formula to calculate the risk value for each patient: risk score = *FADS2* × 0.110 + *KIF20A* × 0.301 + *G6PD* × 0.092. Patients in training cohort (*n* = 345) and validation cohort (*n* = 393) were divided into high-risk and low-risk groups according to the median value of risk scores. The distribution of risk scores, survival status, and prognostic gene expression indicated that patients in the high-risk group had relatively high prognostic gene expression and a higher probability of death than patients in the low-risk group (Figure 2A). PCA, t-SNE and UMAP algorithms were performed to verify the independence of the two clusters, and all three approaches clearly distinguished the high- and low-risk clusters, justifying the grouping (Figures 2B–D). K-M survival analysis revealed that patients in the high-risk group had significantly worse OS than those in the low-risk group (*p* < 0.05, Figure 2E). We wondered whether the risk score of GRFRM was applicable to predict survival in patients with advanced LUAD. 82 patients in TCGA-LUAD were stage III or IV according to the WHO classification. We divided the 82 patients into high- and low-risk groups using optimal cut-off value, and the analysis showed that patients in the high-risk group had worse OS, which was also verified by the late-stage LUAD patients in GSE72094 (Figure 2F). We combined the TCGA-LUAD and GSE97489 cohorts’ patient risk score, age, and tumor stage to construct a nomogram to predict patient survival at 1-, 3-, and 5-year (Figure 2G). Calibration plots showed the accuracy of the nomogram predictions and AUC values corresponding to 1-, 3-, and 5-year survival probabilities were around 0.7, demonstrating the sensitivity and specificity of GRFRM for survival prediction (Figures 2H,I). Thus, these results suggest that the model we constructed shows a significant independent prognostic value.

### The GRFRM is extensively applicable to predict OS of PAAD patients

**FIGURE 3:**
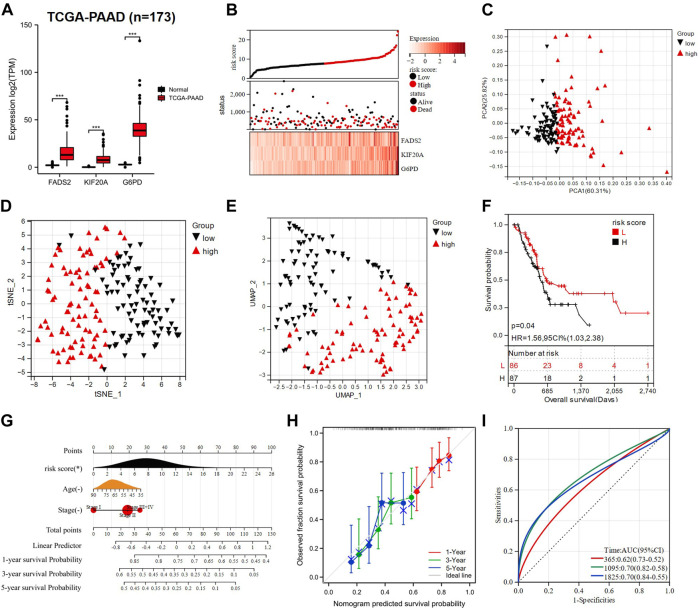
Construction of a PAAD prognostic model based on three candidate genes. **(A)** Comparison of the expression of three prognostic genes in normal (*n* = 167) and PAAD tissues (*n* = 173). **(B)** TCGA-PAAD patients were divided into two groups based on risk scores. **(C–E)** PCA, t-SNE, and UMAP analysis of TCGA-PAAD. **(F)** K-M analysis showed the OS of PAAD patients in the low- and high-risk groups. **(G)** A nomogram was constructed using risk score, age, and tumor stage. **(H)** The calibration curves for predicting PAAD patient OS at 1-, 3- and 5- year. **(I)** ROC analysis of 1-, 3- and 5- year OS in PAAD patients. PAAD: Pancreatic adenocarcinoma; TCGA: The Cancer Genome Atlas; PCA: principal component analysis; t-SNE: t-distributed Stochastic Neighbor Embedding; UMAP: uniform manifold approximation and projection; K-M analysis: Kaplan-Meier analysis; OS: overall survival; CI: combination index; ROC: receiver operating characteristic; AUC: area under the curve; HR: hazard ratio. *p < 0.05; **p < 0.01; ***p < 0.001; ****p < 0.0001; ns *p* > 0.05.

Not limited to LUAD treatment, GEM is also the standard chemotherapy agent for the treatment of late-stage PAAD (Vickers et al., 2012; Okusaka and Furuse, 2020). The induction of ferroptosis in PAAD cells has been shown to potentially give new hope for increasing GEM sensitivity (Tang et al., 2020; Yang J. et al., 2021), so we attempted to explore the applicability of GRFRM in PAAD patients. RNA-seq and clinical information of 173 samples in the TCGA-PAAD cohort were collected. *FADS2*, *KIF20A* and *G6PD* were all highly expressed in PAAD tissues (Figure 3A). The risk scores of each patient in the TCGA-PAAD cohort were calculated as above, and patients were divided into two groups with a median cut-off. More mortality events and higher prognostic gene expression were observed in the high-risk group (Figure 3B), suggesting that the high-risk score reflects the poor prognosis of PAAD patients. Similarly, performing the PCA, t-SNE, and UMAP algorithms validated the grouping (Figures 3C–E), and the K-M survival analysis indicated that patients in the high-risk group had worse OS (*p* < 0.05, Figure 3F). The accuracy of the nomogram used to predict the possibility of survival at 1, 3, and 5 years in patients with PAAD was validated (Figures 3G,H). The AUCs of the ROC at 1-, 3-, and 5-year were 0.62, 0.70, and 0.70, respectively, indicating the high sensitivity and specificity of the prognostic model for OS prediction (Figure 3I). The above analysis illustrates that the GRFRM we constructed is also a good predictor of survival in PAAD patients.

### Somatic mutation and functional enrichment analysis revealed differences between high- and low-risk groups

**FIGURE 4:**
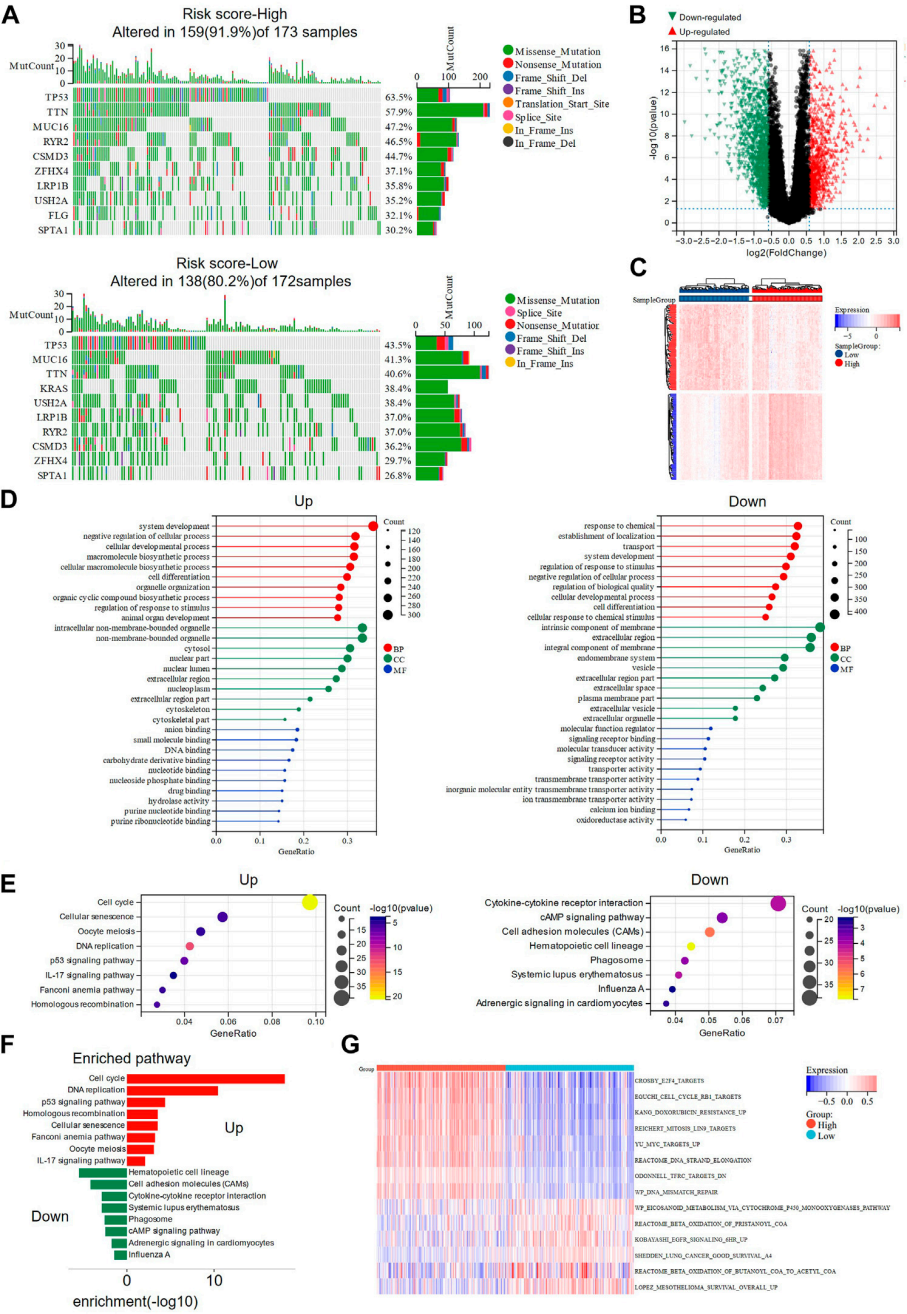
Somatic mutation analysis and functional enrichment analysis of DEGs in high- and low-risk groups. **(A)** The waterfall plots showed the somatic mutation landscape in high and low risk groups. **(B)** Volcano plot presenting the DEGs screened between the high- and low-risk groups. **(C)** The heat map showing the expression of DEGs in high and low-risk groups. **(D)** GO analysis of up-regulated and down-regulated genes in high-risk group were performed separately. **(E,F)** KEGG analysis revealed the main pathways involved in DEGs. **(G)** Pathway activities in high- and low-risk groups were analyzed using GSVA. DEGs: differentially expressed genes; GO: Gene Ontology; KEGG: Genes and Genomes; GSVA: Gene set variation analysis.

Somatic mutation analysis and functional enrichment analysis were performed in high and low-risk groups based on TCGA-LUAD data. Two waterfall plots showed the top 10 mutated genes in the high-risk and low-risk groups (Figure 4A), with *TP53*, *TTN*, and *MUC16* as the most frequently altered genes (top 3) and missense mutations being the common mutation type in both groups. Interestingly, more samples with mutations were observed in the high-risk group (high: 91.9%, low: 80.2%), suggesting that the high-risk score may suggest a higher probability of mutations. In addition, the high-risk group had more patients with TP53 mutations (high: 63.5%, low: 43.5%), which may be associated with ferroptosis and worse survival. A total of 2483 DEGs, including 1059 up-regulated genes and 1424 down-regulated genes, were identified between the high-risk and low-risk groups (|FC|>1.5, *p* < 0.05, Figure 4B, Supplementary Table S2). The heat map showed the distribution of DEGs expression in the high-risk and low-risk groups (Figure 4C). According to the functional enrichment analysis of GO and KEGG pathways, most of the genes in the high-risk group were enriched in signaling pathways such as cell cycle, DNA replication, homologous recombination, and P53, indicating that the high-risk group may have stronger DNA damage repair ability, which is a factor of drug resistance (Pilie et al., 2019) (Figures 4D–F). According to the GSVA results, the high-risk group was enriched in drug resistance-related pathways, while fatty acid metabolism-related pathways (Shi and Tu, 2015) and pathways beneficial to lung cancer survival were highly enriched in the low-risk group (Figure 4G). Interestingly, enrichment of the EGFR pathway was also presented in the low-risk group, and its activation was shown to sensitize NSCLC cells to ferroptosis (Poursaitidis et al., 2017).

### GRFRM suggests differences in anticancer drugs sensitivity in LUAD

**FIGURE 5:**
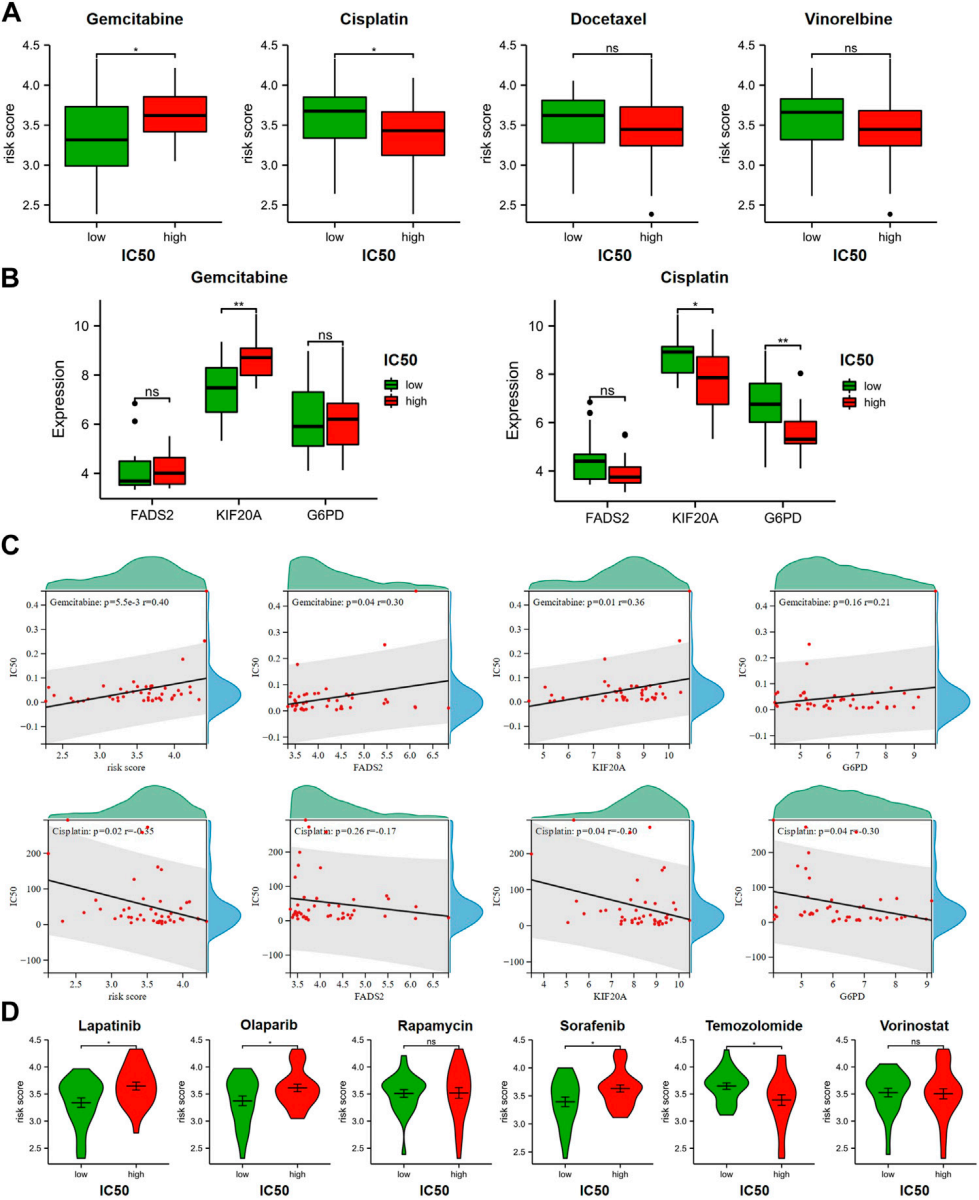
Relationship between GRFRM risk score and drug sensitivity. **(A)** Correlation analysis of IC50 and risk scores for GEM and chemotherapeutic agents commonly combined with GEM for LUAD. **(B)** Comparison of the expression levels of three prognostic genes in GEM and Cisplatin high and low IC50 groups, respectively. **(C)** Spearman coefficients were used for correlation analysis of drug IC50 and risk scores, as well as correlation analysis of drug IC50 and expression levels of three candidate genes. **(D)** Drug sensitivity of 4 FINs available for LUAD treatment was associated with GRFRM risk scores. GRFRM: GEM response and ferroptosis related model; GEM: Gemcitabine; LUAD: lung adenocarcinoma; IC50: half maximal inhibitory concentration; FINs: ferroptosis inducers. *p < 0.05; **p < 0.01; ns *p* > 0.05.

Based on the IC50 of various anticancer agents obtained from GDSC for LUAD cell lines, we tried to explore the relationship between this GRFRM and drug sensitivity and the potential treatment mode for LUAD. GEM, Cisplatin and Docetaxel, and Vincristine are common chemotherapeutic agents used alone or in combination for LUAD (Thai et al., 2021), and we compared the risk scores of LUAD cell lines in the high IC50 and low IC50 groups for the four drugs, respectively (Figure 5A). The IC50 of GEM was higher in LUAD cell lines with higher risk scores, indicating that the decrease in risk was accompanied by an increase in sensitivity to GEM, which is consistent with our original intention of constructing a risk model. However, the opposite was true for Cisplatin, which may suggest that GEM may be a good choice in the case of insensitivity to Cisplatin therapy. The analysis of Docetaxel and Vinorelbine was not statistically different and therefore will not be further explored. Besides, we found that the expression levels of *FADS2* and *KIF20A* were lower in GEM-sensitive cell lines, while the expression levels of *KIF20A* and *G6PD* were higher in Cisplatin-sensitive cell lines (Figure 5B). The above results were also verified using spearman correlation analysis (Figure 5C). Thus, we conclude that *KIF20A* is a core gene in this model and its high expression level is associated with this relatively high-risk score, as well as low GEM sensitivity and high Cisplatin sensitivity. Cisplatin, Lapatinib, Olaparib, Rapamycin, Sorafenib, Temozolomide (TMZ) and Vorinostat are reported FINs that are also available for LUAD therapy. Compared to the high-risk group, Lapatinib, Olaparib and Sorafenib had lower IC50 values in low-risk group, and these drugs may be able to produce a favorable effect in combination with GEM. Brain metastases are one of the most common events in advanced lung cancer, and TMZ is effective in brain metastasized NSCLC (Tsakonas et al., 2017). Here, the results of the TMZ analysis suggest a different message than the above drugs, and we suspect that as the risk score increases, the increased sensitivity of TMZ may be good for the therapy of LUAD in the case of brain metastases (Figure 5D).

### GEM and IKE synergistically inhibit the proliferation of LUAD cells

**FIGURE 6:**
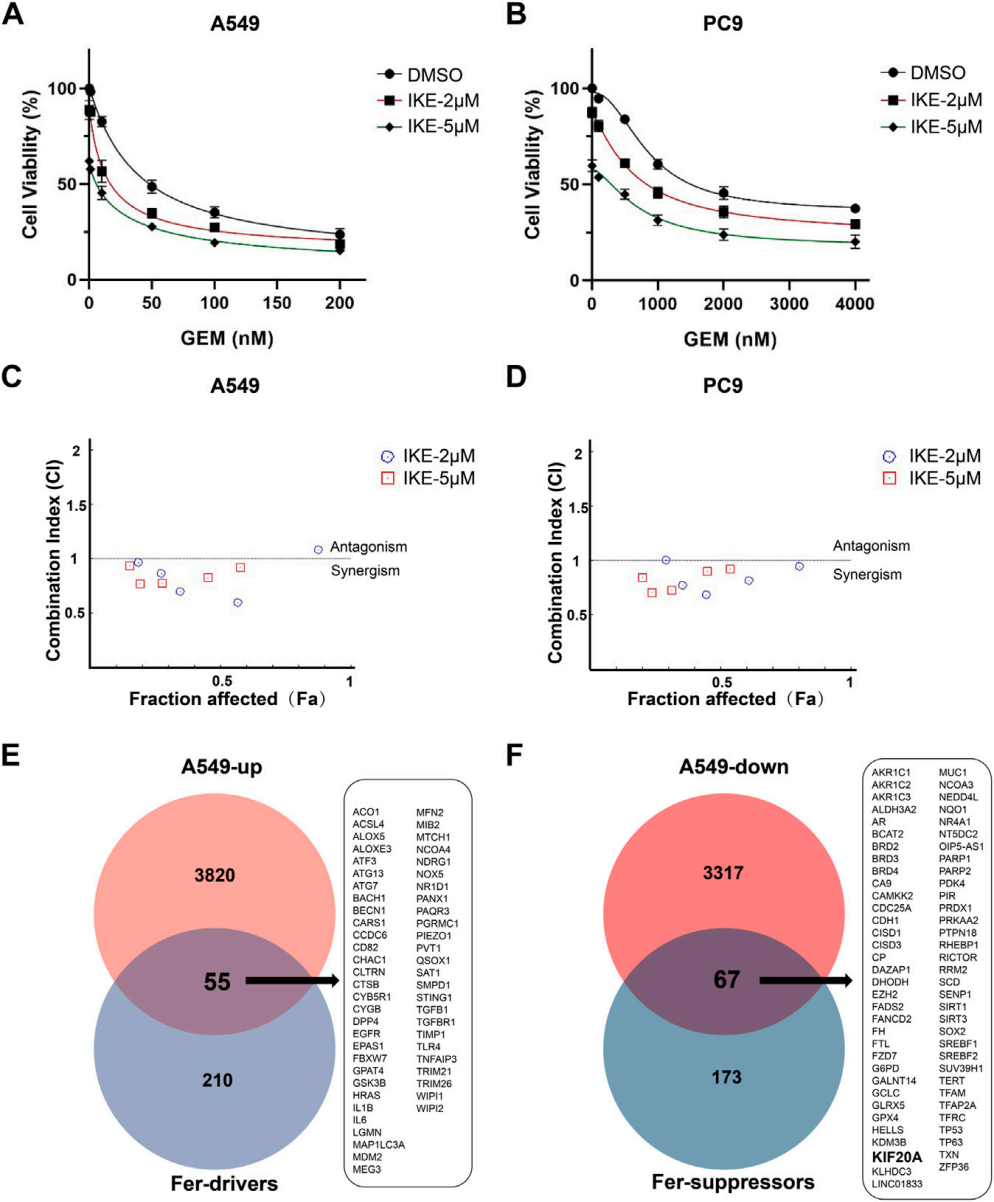
Combined treatment of GEM and IKE synergistically inhibited the proliferation of A549 and PC9. **(A)** A549 cell viability was assessed by CCK8 assay after treatment with different concentrations of GEM alone (0, 10, 50, 100 or 200 nM) or in combination with IKE (2 or 5 μM) for 48 h **(B)** PC9 cell viability was assessed by CCK8 assay after treatment with different concentrations of GEM alone (0, 100, 500, 1000 or 2000 nM) or in combination with IKE (2 or 5 μM) for 48 h **(C,D)** The software calculated and evaluated the combination index (CI) of GEM and IKE in A549 and PC9. CI = 1, additive; CI > 1, antagonism; CI < 1, synergism. **(E)** Venn diagram showing ferroptosis driver genes upregulated by GEM treatment in A549. **(F)** Venn diagram showing ferroptosis suppressors downregulated by GEM treatment in A549. *n* = 3. GEM: Gemcitabine; IKE: Imidazole Ketone Erastin; CCK8 assay: Cell Counting Kit-8 assay; CI: combination index.

GEM and the ferroptosis inducer IKE have been proved to be effective in inhibiting the proliferation of LUAD cells (Wang et al., 2021; Xiang et al., 2022), but it is not clear how the combination of the two works. LUAD cells A549 and PC9 were treated with different concentrations of GEM (0–200 nM/0–4000 nM) in combination without or with IKE (2 μM or 5 μM) for 48 h and cell proliferation was analyzed by the CCK8 assay. Our results showed that both 2 μM and 5 μM concentrations of IKE were effective in inhibiting the proliferation of A549 and PC9 cells in combination with GEM, and this inhibition had GEM concentration-dependent (Figure 6A,B). The CI was calculated and visualized by CompuSyn software. CI plots showed that most CI values were less than 1 in A549 and PC9 cells (Figure 6C,D), indicating a synergistic decrease in cell viability following the combination of GEM and IKE. To investigate whether this synergistic effect was associated with further enhancement of ferroptosis, we analyzed the RNA-seq data of A549 treated with GEM in combination with FRGs. The results showed that 55 ferroptosis driver genes including *ALOX5*, *ACSL4* were up-regulated and 67 suppressors including *KIF20A*, *FADS2*, *G6PD, SLC7A11*, *GPX4* were down-regulated in A549 after GEM treatment (Figure 6E,F). Thus, the change of FRGs expression may be the reason for the synergistic effect of GEM ferroptosis inducers IKE.

### Knockdown of *KIF20A* enhanced the combined effect of GEM and IKE

**FIGURE 7:**
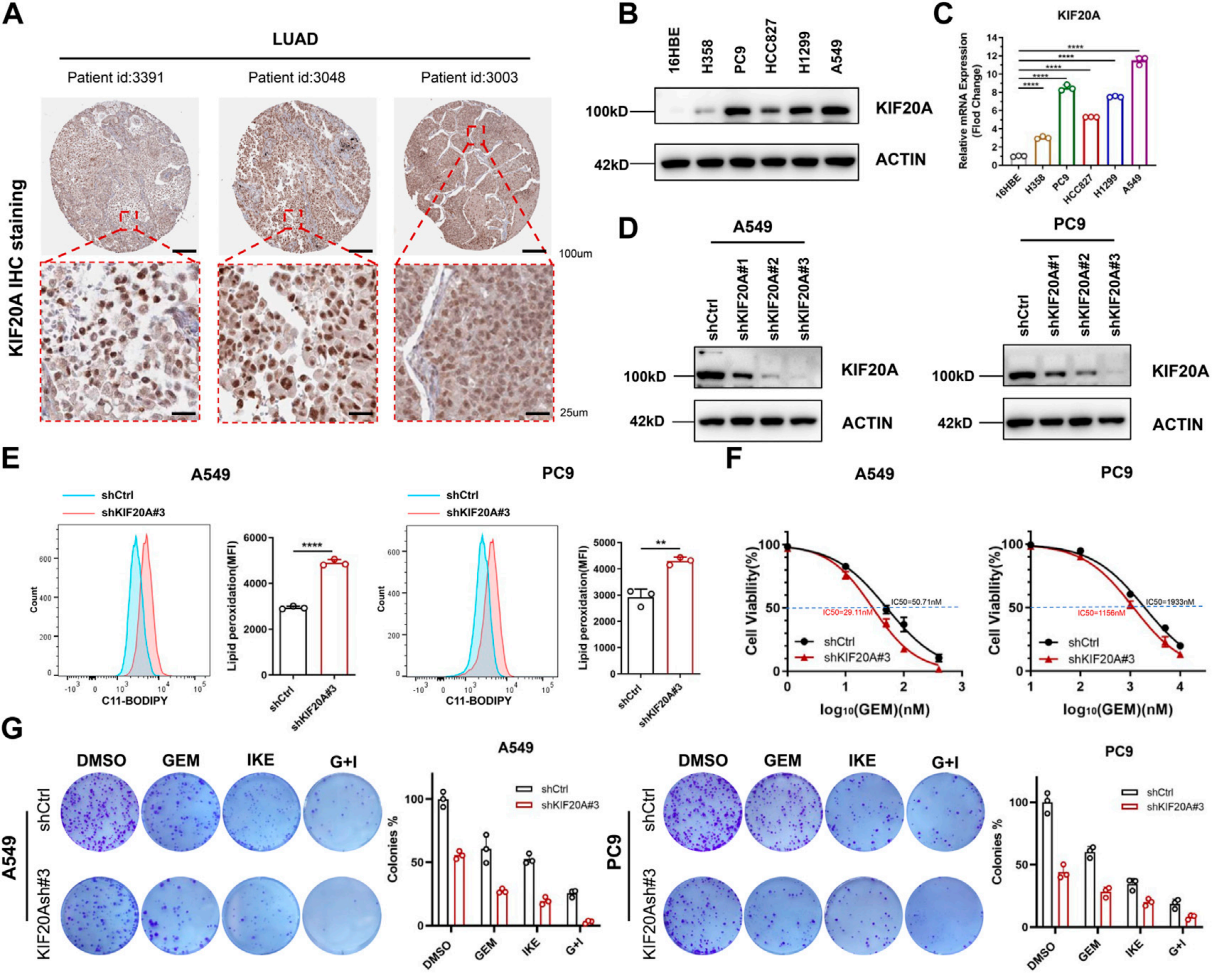
KIF20A was highly expressed in LUAD cells and regulated the combined effect of GEM and IKE. **(A)** IHC staining of KIF20A protein in LUAD tissues was analyzed based on the HPA database. **(B)** Western blot analysis was used to detect KIF20A protein levels in human bronchial epithelial-like cells (16HBE) and LUAD cell lines (H358, PC9, HCC8217, H1299, A549). **(C)** RT-qPCR for detection of KIF20A mRNA levels in 16HBE and LUAD cell lines. **(D)** Western blot analysis for detecting knockdown of KIF20A in PC9 and A549. **(E)** Lipid ROS production was measured by flow cytometry using C11-BODIPY. A549/PC9 cells were treated with IKE for 24 h **(F)** CCK8 assay to detect the IC50 value of GEM in A549/PC9 after KIF20A knockdown. **(G)** Colony-forming Assay to assess the effect of KIF20A knockdown on the combined IKE and GEM. *n* = 3. LUAD: lung adenocarcinoma; GEM: Gemcitabine; IHC: Immunohistochemical; RT-qPCR: Real-Time Quantitative PCR; ROS: reactive oxygen species; IKE: Imidazole Ketone Erastin; IC50: half maximal inhibitory concentration; CCK8 assay: Cell Counting Kit-8 assay; G + I: Gemcitabine + IKE. *p < 0.05; **p < 0.01; ***p < 0.001; ****p < 0.0001; ns *p* > 0.05.

Based on above results, *KIF20A* is a key gene in this prognostic model and has a potential role in regulating the combination of GEM and IKE. Additionally, *KIF20A* has been shown to promote proliferation of LUAD cells and mediate colorectal cancer (CRC) resistance to Oxaliplatin by inhibiting ferroptosis (Zhao et al., 2018; Yang C. et al., 2021). We first downloaded IHC images (https://www.proteinatlas.org/ENSG00000112984-KIF20A/pathology/lung+cancer#) from the HPA database and observed that KIF20A stained positive and generally strong in LUAD tissues, mainly located in the nuclear of LUAD cells (Figure 7A). Compared with 16HBE, KIF20A was highly expressed in A549, PC9 and H1299 cell lines (Figures 7B,C), so we selected A549 and PC9 for *KIF20A* knockdown and verified the knockdown effect by western blot analysis (Figure 7D), with sh*KIF20A*#3 selected for subsequent experiments. Production of lipid ROS is one of the classical features of ferroptosis occurrence. We found that the depletion of KIF20A increased lipid ROS in A549 and PC9 cells after being treated with IKE for 24 h (Figure 7E), suggesting that knockdown of *KIF20A* may have increased IKE-induced ferroptosis. In addition, CCK8 experiments demonstrated that knockdown of *KIF20A* resulted in PC9 and A549 being more sensitive to GEM (Figure 7F). IKE inhibited LUAD cell colony formation when used alone and significantly inhibited cell proliferation when co-treated with GEM (Figure 7G). More interestingly, knockdown of *KIF20A* enhanced the anti-proliferative effects of GEM and IKE alone and in combination (Figure 7G). Taken together, these results suggest that combined treatment with GEM and IKE has a strong synergistic anticancer effect on LUAD cells and that knockdown of *KIF20A* enhances this effect, thus *KIF20A* may be an essential linker between GEM response and ferroptosis in LUAD cells.

## Discussion

Since the discovery of the novel RCD ferroptosis, a growing number of studies have shown that ferroptosis induction significantly inhibits cancer progression, and it is expected to bring hope for the treatment of cancer with apoptosis defects (Friedmann Angeli et al., 2019; He et al., 2021). Patients who have a defect in intrinsic sensitivity or downregulated extrinsic sensitivity of GEM may also benefit from the induction of ferroptosis. Glutathione peroxidases 4 (GPX4) catalyzes the reduction of cellular lipid peroxidation to avoid its deleterious effects and is considered to be a central inhibitor of ferroptosis (Yang et al., 2014). Zhu et al. (2017) revealed that the heat shock 70 kDa protein 5 (HSPA5) interacts with GPX4 protein in pancreatic ductal adenocarcinoma cells to inhibit ferroptosis, and they observed that inhibition of the HSPA5-GPX4 pathway enhanced the sensitivity of GEM *in vitro* and *in vivo*. Acyl-CoA synthetase long-chain family member 4 (ACSL4) acts as a lipid metabolizing enzyme to add coenzyme A to arachidonic acid and positively regulates ferroptosis occurrence (Chen et al., 2021b). Ye et al. (2020) showed that knockdown of ADP Ribosylation Factor 6 can enhance RSL3-induced ferroptosis by regulating ACSL4, which partially enhanced the sensitivity of PAAD cells to GEM. In addition, GEM can also induce the accumulation of ROS, which may further induce the development of ferroptosis (Ju et al., 2015). Recent reports on biomaterials suggest that GEM-loaded carbonaceous nanoparticles can enhance the synergistic anticancer effects of ferroptosis and GEM chemotherapy (Zhang et al., 2022). However, the above studies are limited to PAAD, and robust evidence on the association between ferroptosis and GEM response in LUAD is not available to illustrate.

Based on RNA-seq of GEM-treated A549, GDSC data, TCGA cohort and GEO dataset, we originally screened FRGs with survival prognostic features and correlated with GEM sensitivity. Three identified candidate genes, *FADS2*, *KIF20A*, and *G6PD*, were highly expressed in LUAD tissues and their high expression correlated with poor patient prognosis, and tumor stage. The fatty acid desaturase 2 (FADS2) is a rate-limiting enzyme in polyunsaturated fatty acid (PUFA) desaturation and can regulate GPX4 expression (Park et al., 2015; Xuan et al., 2022). Researchers observed increased iron levels and lipid ROS in A549 cells with knockdown of *FADS2*, as well as more marked erastin-induced cell death events (Jiang et al., 2017). The enzyme glucose-6-phosphate dehydrogenase (G6PD) is involved in the maintenance of redox homeostasis as a catalytic enzyme of the oxidative pentose phosphate pathway and may be a therapeutic target for cancers (Yang et al., 2019; Ghergurovich et al., 2020). Kinesin family member 20A (KIF20A) is engaged in the cytoplasmic division, organelle transport and cell-directed motility (Taniuchi et al., 2005; Mandal et al., 2019; Wu et al., 2019). Overexpression of *KIF20A* has been reported to be associated with cancer progression and chemoresistance in NSCLC, CRC and HCC (Zhao et al., 2018; Xie et al., 2020; Wu et al., 2021) Yang C. et al. (2021) reported that *KIF20A* induced *NUAK1* activation upregulating *GPX4* levels, which maintained intracellular redox homeostasis and inhibited ferroptosis, ultimately leading to CRC resistance to oxaliplatin. More interestingly, in a phase I clinical trial, researchers combined a KIF20A-derived peptide with GEM as a novel immunotherapeutic agent to treat advanced PAAD patients and achieved longer overall survival, suggesting the role of *KIF20A* as an anticancer therapeutic target and the possibility of modulating GEM sensitivity (Suzuki et al., 2014). Before this study, it was unknown whether *KIF20A* could affect the sensitivity of LUAD cells to GEM by regulating ferroptosis. We demonstrate that knockdown of *KIF20A* enhanced induced ferroptosis and the sensitivity of LUAD cells to GEM, and GEM in combination with the IKE which can induce ferroptosis also had a synergistically anti-proliferation effect, which was further enhanced in the presence of *KIF20A* knockdown.

A risk model constructed from three candidate genes divides LUAD patients into low- and high-risk scoring groups with different OS, making it easier to use ferroptosis regulators to predict survival outcomes in LUAD patients. Patients in the high-risk group had worse overall survival than those in the low-risk group, and the same results were also seen in the group of patients with advanced LUAD. Compared to the low-risk group, there were more patients with somatic mutations in the high-risk group. *TP53* mutations affect ferroptosis-related metabolism and are detrimental to the survival of cancer patients (Jiang et al., 2015), and we observed the presence of *TP53* somatic mutations in 63.5% of patients in the high-risk group. In addition, KEGG and GSVA analysis revealed that ferroptosis-related signaling pathways such as P53, GEM anti-cancer mechanism-related signaling pathways such as DNA replication, and cellular recycling pathways were significantly enriched in the high-risk group, while fatty acid metabolic signaling pathways were enriched in the low-risk group. These results provide further evidence for differences in ferroptosis and GEM response between the two groups. Besides, drugs sensitivity analysis showed that GEM, Lapatinib, Olaparib, and Sorafenib are ideal for LUAD patients in the low-risk score group, while Cisplatin treatment was the opposite, which may guide different treatment modalities in the two groups. For example, FIN lapatinib combined with GEM may achieve a triple anti-cancer effect of targeted therapy, chemotherapy therapy, and induction therapy of ferroptosis in a low-risk group (Oh and Bang, 2020; Su et al., 2020).

In contrast to other reported ferroptosis-related prognostic models, we introduced the requirement of drug sensitivity correlation in the screening of genes, which emphasizes the intrinsic relationship between ferroptosis and drug sensitivity and makes the GRFRM a more powerful guide for drug use. Some limitations of our study still exist. The effectiveness of the constructed model needs to be further validated by other clinical studies in the future. Further experiments to validate and analyze the relationship between *KIF20A*, ferroptosis, and GEM response are also necessary.

## Conclusion

In conclusion, we developed and validated a signature with three FRGs for probing the relationship between ferroptosis and GEM response and predicting OS of LUAD patients, and our study demonstrated that GEM and the ferroptosis inducer IKE synergistically inhibited the proliferation of LUAD cells. Targeting the FRG *KIF20A* can enhance ferroptosis and modulate the combination of GEM and IKE, which might serve as a therapeutic target in LUAD.

## Data Availability

Publicly available datasets were analyzed in this study. This data can be found here: GDSC: https://www.cancerrxgene.org/, TCGA: https://cancergenome.nih.gov/, GEO: http://www.ncbi.nlm.nih.gov/geo, FerrDb: http: //www.zhounan.org/ferrdb/current/, GTEx: https://www.gtexportal.org, HPA: https://www.proteinatlas.org/.

## Glossary

NSCLC: non-small cell lung cancer
LUAD: lung adenocarcinoma
GEM: Gemcitabine
ROS: reactive oxygen species
RCD: regulated cell death
FINs: ferroptosis inducers
HCC: Hepatocellular carcinoma
SLC7A11: solute carrier family seven member 11
PAAD: Pancreatic adenocarcinoma
FRGs: ferroptosis-related genes
FBS: fetal bovine serum
RNA-seq: RNA sequencing
TRIzol: Total RNA Extraction Reagent
DEGs: differentially expressed genes
IC50: half maximal inhibitory concentration
GDSC: Drug Sensitivity in Cancer
TCGA: The Cancer Genome Atlas
GEO: Gene Expression Omnibus
GTEx: Genotype-Tissue Expression
IHC: Immunohistochemical
HPA: Human Protein Atlas
HR: hazard ratio
OS: overall survival
t-SNE: t-distributed Stochastic Neighbor Embedding
UMAP: uniform manifold approximation and projection
PCA: principal component analysis
K-M survival analysis: Kaplan-Meier survival analysis
ROC: receiver operating characteristic
AUC: area under the curve
GO: Gene Ontology
KEGG: Kyoto Encyclopedia of Genes and Genomes
GSVA: Gene set variation analysis
RT-qPCR: Real-Time Quantitative PCR
CCK8 assay: Cell Counting Kit-8 assay
IKE: Imidazole Ketone Erastin
CI: combination index
GRFRM: GEM response and ferroptosis related model
CRC: colorectal cancer
TMZ: Temozolomide
GPX4: glutathione peroxidases four
HSPA5: heat shock 70 kDa protein five
ACSL4: Acyl-CoA synthetase long-chain family member four
FADS2: fatty acid desaturase two
PUFA: polyunsaturated fatty acid
G6PD: glucose-6-phosphate dehydrogenase
KIF20A: Kinesin family member 20A
