# Supplementary material for: KIF20A is associated with clinical prognosis and synergistic effect of gemcitabine combined with ferroptosis inducer in lung adenocarcinoma
Source: Front Pharmacol. 2022 Sep 26;13:1007429. doi: 10.3389/fphar.2022.1007429 (PMC9549118; doi:10.3389/fphar.2022.1007429)
Supplement: Supplementary file 1 [file DataSheet1.docx]

Supplementary Material

**Supplementary Table 1 Results of the multifactorial Cox regression analysis of FRGs in TCGA-LUAD and GSE72094 (top 15 in HR ranking)**

**Ferroptosis Driver in TCGA-LUAD**

| Features | p-value | Hazard Ratio(95%CI) |
| --- | --- | --- |
| IFNA4 | 0.0029 | 30.22(2.22-411.20) |
| IFNA8 | 0.07 | 1.97(0.94-4.12) |
| CFL1 | 0.00150 | 1.91(1.28-2.85) |
| IFNA13 | 0.002 | 1.82(1.24-2.68) |
| ELAVL1 | 0.1 | 1.63(0.91-2.92) |
| IFNA1 | 0.03 | 1.63(1.03-2.57) |
| AGPS | 0.01 | 1.58(1.11-2.25) |
| VDAC2 | 0.0015 | 1.58(1.19-2.10) |
| IFNA14 | 0.61 | 1.51(0.31-7.46) |
| NRAS | 0.0054 | 1.46(1.12-1.91) |
| PANX1 | 0.01 | 1.44(1.08-1.94) |
| HMGB1 | 0.03 | 1.43(1.03-1.98) |
| DLD | 0.05 | 1.40(1.00-1.96) |
| TBK1 | 0.09 | 1.38(0.95-2.00) |
| C19orf61 | 0.09 | 1.36(0.95-1.93) |

**Ferroptosis Suppressor in TCGA-LUAD**

| Features | p-value | Hazard Ratio(95%CI) |
| --- | --- | --- |
| FTMT | 0.16 | 1.83(0.76-4.36) |
| AKT1S1 | 0.0014 | 1.72(1.24-2.40) |
| VDAC2 | 0.0015 | 1.58(1.19-2.10) |
| VCP | 0.05 | 1.52(1.00-2.33) |
| ACSL3 | 0.00093 | 1.48(1.17-1.86) |
| RELA | 0.14 | 1.47(0.88-2.47) |
| DAZAP1 | 0.11 | 1.46(0.92-2.31) |
| TFAM | 0.02 | 1.45(1.05-2.00) |
| SLC3A2 | 0.006 | 1.43(1.11-1.85) |
| SFRS9 | 0.04 | 1.43(1.02-2.00) |
| KIF20A | 0.0000046 | 1.41(1.22-1.64) |
| ARF6 | 0.05 | 1.41(1.00-1.99) |
| TMEM189 | 0.03 | 1.39(1.04-1.86) |
| NEDD4 | 0.00097 | 1.38(1.14-1.67) |
| COPZ1 | 0.12 | 1.36(0.92-2.02) |

**Ferroptosis Driver in GSE72094**

| Features | p-value | Hazard Ratio(95%CI) |
| --- | --- | --- |
| VDAC2 | 0.000001 | 3.50(2.13-5.74) |
| DNAJB6 | 0.0064 | 2.67(1.32-5.40) |
| ATP5G3 | 0.03 | 2.62(1.12-6.11) |
| HIF1A | 0.02 | 2.02(1.13-3.62) |
| KRAS | 0.02 | 1.97(1.12-3.47) |
| MAPK8 | 0.03 | 1.96(1.06-3.63) |
| EPT1 | 0.0018 | 1.89(1.27-2.82) |
| PANX1 | 0.0051 | 1.74(1.18-2.57) |
| TIMM9 | 0.05 | 1.74(1.00-3.03) |
| DLD | 0.08 | 1.70(0.94-3.07) |
| WIPI2 | 0.1 | 1.65(0.92-2.97) |
| CFL1 | 0.17 | 1.65(0.81-3.35) |
| C7orf68 | 0.000000022 | 1.64(1.38-1.96) |
| LIG3 | 0.02 | 1.63(1.09-2.44) |
| CBARA1 | 0.1 | 1.57(0.91-2.71) |

**Ferroptosis Suppressor in GSE72094**

| Features | p-value | Hazard Ratio(95%CI) |
| --- | --- | --- |
| VDAC2 | 0.000001 | 3.50(2.13-5.74) |
| RELA | 0.02 | 2.41(1.17-4.95) |
| TMEM189 | 0.0004 | 2.30(1.45-3.65) |
| HIF1A | 0.02 | 2.02(1.13-3.62) |
| PPARD | 0.02 | 1.96(1.10-3.49) |
| PRDX1 | 0.11 | 1.81(0.88-3.69) |
| CISD1 | 0.01 | 1.79(1.13-2.83) |
| AKT1S1 | 0.01 | 1.73(1.13-2.64) |
| AIFM2 | 0.0022 | 1.72(1.22-2.44) |
| SLC3A2 | 0.03 | 1.63(1.05-2.52) |
| ARF6 | 0.15 | 1.59(0.84-3.00) |
| PRDX6 | 0.2 | 1.58(0.79-3.15) |
| TXN | 0.0055 | 1.58(1.14-2.18) |
| BRD3 | 0.03 | 1.58(1.05-2.37) |
| ATF4 | 0.57 | 1.56(0.34-7.11) |

**Supplementary Figure S1: Expression of FADS2, KIF20A and G6PD in LUAD samples with different TNM stages.**

**
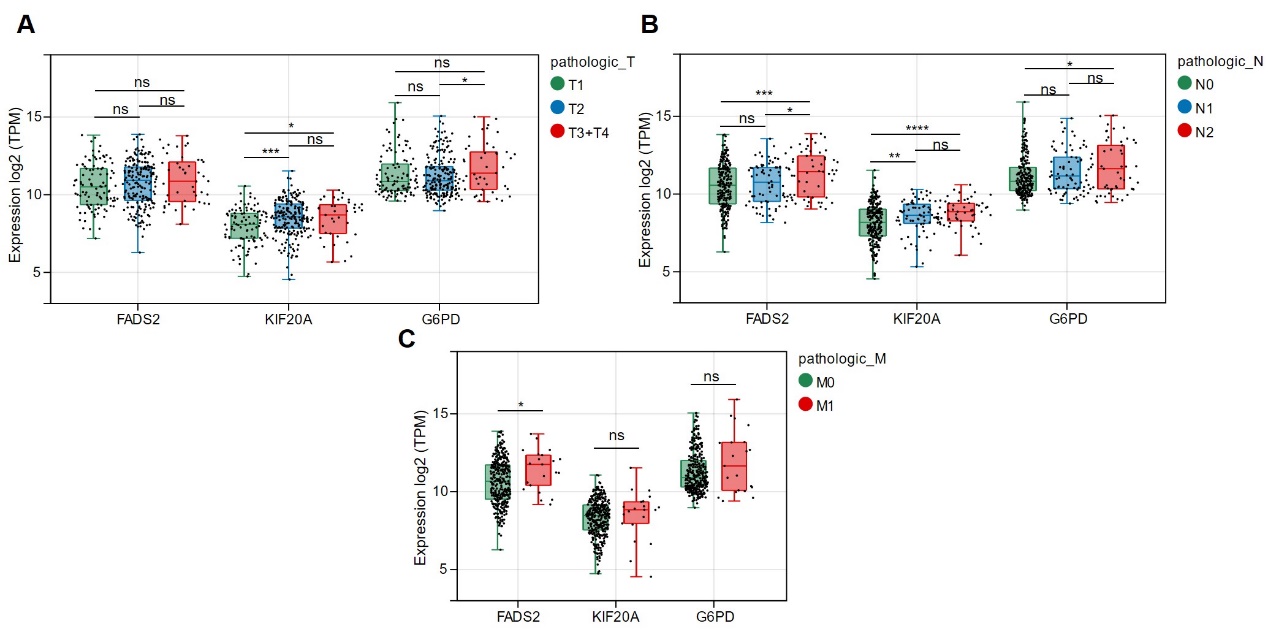
**

**Supplementary Figure 1.** Comparing the expression of three prognostic genes in LUAD with different TNM stages.
